# The adsorption routes of 4IR technologies for effective desulphurization using cellulose nanocrystals: Current trends, challenges, and future perspectives

**DOI:** 10.1016/j.heliyon.2024.e24732

**Published:** 2024-01-18

**Authors:** Oluwagbenga A. Olawuni, Olawumi O. Sadare, Kapil Moothi

**Affiliations:** a Department of Chemical Engineering, Faculty of Engineering and the Built Environment, University of Johannesburg, Doornfontein Campus, Johannesburg, 2028, South Africa; b Department of Chemical Engineering, Water Innovation and Research Centre (WIRC), University of Bath, Claveton Down, Bath, North East Somerset, BA27AY, South West, United Kingdom; c School of Chemical and Minerals Engineering, Faculty of Engineering, North-West University, Potchefstroom, 2520, South Africa

**Keywords:** Adsorptive desulphurization, Artificial intelligence, Green isolation, Nanocellulose crystal, Environmental sustainability

## Abstract

The combustion of liquid fuels as energy sources for transportation and power generation has necessitated governments worldwide to direct petroleum refineries to produce sulphur-free fuels for environmental sustainability. This review highlights the novel application of artificial intelligence for optimizing and predicting adsorptive desulphurization operating parameters and green isolation conditions of nanocellulose crystals from lignocellulosic biomass waste. The shortcomings of the traditional modelling and optimization techniques are stated, and artificial intelligence's role in overcoming them is broadly discussed. Also, the relationship between nanotechnology and artificial intelligence and the future perspectives of fourth industrial revolution (4IR) technologies for optimization and modelling of the adsorptive desulphurization process are elaborately discussed. The current study surveys different adsorbents used in adsorptive desulphurization and how biomass-based nanocellulose crystals (green adsorbents) are suitable alternatives for achieving cleaner fuels and environmental sustainability. Likewise, the present study reports the challenges and potential solutions to fully implementing 4IR technologies for effective desulphurization of liquid fuels in petroleum refineries. Hence, this study provides insightful information to benefit a broad audience in waste valorization for sustainability, environmental protection, and clean energy generation.

## Highlights

• Adsorptive desulphurization of liquid fuels to promote environmental sustainability.
• 4IR technologies application to improve the desulphurization processes.
• Valorization of biomass waste to generate value-added products.
• Green synthesis strategies of nanocellulose crystal from waste biomass.
• Nanotechnology and artificial intelligence to boost the production of cleaner fuels.

## 1 Introduction

The annual global energy demand trend revealed an increased demand for energy in three major economic sectors: industrial, residential, and transportation. Despite the environmental pollution from using fossil fuels (crude oil, natural gas, coal), they still make up a significant share of global energy consumption [1]. Crude oil is the most extensively utilized fuel for transportation. Hence, petroleum refineries are indirectly and directly perceived as significant environmental polluters [2]. As a result, petroleum refineries are mandated to produce cleaner fuels with reduced sulphur content. For instance, in South Africa, the use of fossil fuel as an energy source is over 87 %, of which 80 % is consumed in transportation [3]. The presence of active organic sulphur compounds in gasoline has resulted in the deactivation of the catalytic converter, corrosion of metallic components, air pollution, environmental pollution, and formation of acid rain [4,5].

Sulphur–rich liquid fuels emit significant quantities of sulphur oxides (SO_x_) into the atmosphere during combustion, resulting in acid rain and environmental pollution that is detrimental to human health [5]. Similarly, high levels of organic sulphur compounds in fuel oil have led to severe corrosion issues in refining machinery and the deactivation of some catalysts in automotive catalytic converters [6]. Due to the serious environmental concerns caused by the combustion of liquid fuels for transportation and power generation, governments across the globe have given stringent regulations to lessen the discharge of sulphur compounds into the environment from the utilization of liquid fuels, and petroleum refineries are instructed to comply with these regulations [2]. This review explored the application of the fourth industrial revolution (4IR) technologies to the adsorptive desulphurization of refinery products to attain the globally acceptable sulphur level of ≤10 ppm.

The Fourth Industrial Revolution (4IR) combines technology that distorts previously independent technologies between the digital, biological, and physical realms. The 4IR refers to cutting-edge technologies such as artificial intelligence (AI), Internet of Things (IoT), virtual reality (3D printing), nanomaterials, big data, blockchain, and biotechnology that are critical tools for ensuring the sustainability of the future [7,8]. Artificial intelligence (AI), especially machine learning techniques, designs and models algorithms that enable the computer to automatically learn and predict the relationship between dependent and independent variables [9,10]. The simulation and modelling of the desulphurization process have been suggested in various studies to aid in a better understanding of the process activities. Besides, machine learning techniques are applied to predict multiple methods to save cost, time, and energy. Adaptive neuro-fuzzy interference systems (ANFIS), artificial neural networks (ANN), support vector machines (SVM), fuzzy logics, and genetic algorithms (GA) are some examples of machine learning techniques that are widely applied to optimize and predict industrial processes [9,11].

The optimization of the desulphurization process is mainly based on experimental values collected to develop empirical and optimization models [12], and most of the studies in this area focused on the hydrodesulphurization process. Al-Jamimi et al. [12] reviewed several studies on optimizing hydrodesulphurization using machine learning (ML) techniques. However, the hydrodesulphurization is unable to achieve the global standard of sulphur content (≤10 ppm) in refinery products, and the process is expensive because this desulphurization technique involves a catalytic reaction of hydrogen with sulphur organic compounds in liquid fuels at elevated temperature (320–360 °C), and pressure (10–90 bar) to produce hydrogen sulphide that is later removed from liquid fuels [13]. Therefore, there is significant interest in utilizing ML models to predict the optimal operating conditions for desulphurization, find fossil fuel leaks from oil refineries, transport and collect natural gas, and reduce CO_2_ emissions by carbon capture and sequestration.

This review aims to explore and outline the application of 4IR technologies in the adsorptive desulphurization of liquid fuels. The relationship between nanotechnology and artificial intelligence, the future perspectives of 4IR technologies for desulphurization of liquid fuels, and their roles in achieving cleaner fuels are highlighted in this review. Furthermore, the recent development in artificial intelligence applications for nanocellulose crystals extraction and the adsorptive desulphurization process is discussed. Also, the current issues and potential prospects for using artificial intelligence to build cutting-edge nanocellulose crystal extraction and adsorptive desulphurization systems are reported. The review offers valuable insight for researchers to develop precise scientific formulations and plan experiments for further studies on environmentally friendly isolation methods and artificial intelligence-assisted adsorptive desulphurization.

## 2 The fourth industrial revolution (4IR) technologies for desulphurization of organic sulphur compounds

### 2.1 Relationship between nanotechnology and artificial intelligence

**Fig. 1:**
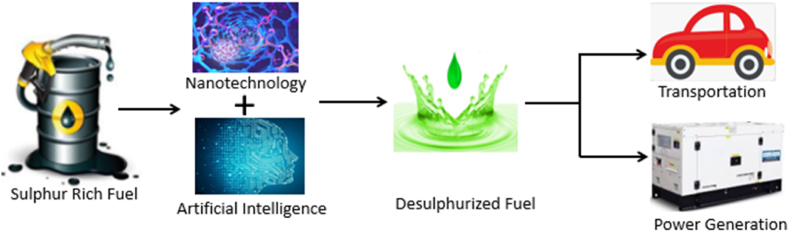
Relationship between nanotechnology and artificial intelligence for desulphurization.

Nanotechnology integrates the principles of chemistry, physics, and engineering for different purposes, while artificial intelligence uses the human neural system to predict, optimize, and model the outcomes of scientific processes [[14], [15], [16]]. Artificial intelligence has been applied to develop numerical simulations, analytical approximations, and accurate interpretations of complex and experimental data. Because of this, artificial intelligence can be merged with nanotechnology to generate and scrutinize scientific data accurately for the advancement of nano-applications that will significantly improve our society and promote the Industrial Revolution. In addition, enhancing nano-applications such as nano-computing, nano-devices, and nanomaterials has increased the power of artificial intelligence tools and shown the synergetic relationship between nanotechnology and artificial intelligence [14,15]. Moreover, this review focused on the integration of nanotechnology and artificial intelligence to solve complex engineering problems, reduce errors associated with the size of materials or systems, and use the data obtained from adsorptive desulphurization systems to predict the behaviour of the systems and optimize the operating conditions. Additionally, combining nanotechnology and artificial intelligence, as seen in Fig. 1, would boost the desulphurization process of liquid fuels to achieve the globally acceptable sulphur level (<10 ppm), and this review elaborately discussed the novel approach of applying nanotechnology and artificial intelligence in the following sections to achieving cleaner fuel for environmental sustainability.

### 2.2 Application of fourth industrial revolution (4IR) technologies for desulphurization of liquid fuels

Artificial intelligence (AI) and nanotechnology are part of the fourth industrial revolution (4IR) technologies that have been relatively deployed in the desulphurization of liquid fuels. The technologies are essential for improving cleaner fuels and a sustainable environment. Recently, there has been remarkable development and broad applications of nanomaterial/nanotechnology in industrial processes [8]. Likewise, artificial intelligence via machine learning techniques has been applied for modelling and predicting different industrial processes. Machine learning approaches have recently been created and successfully used to tackle various nonlinear engineering problems, raising interest in their potential applications in other fields [17]. Due to the usage of intelligent systems, they have grown since they are practical tools for modelling processes in the petroleum industry [18]. Therefore, this paper will examine how machine-learning tools model and optimize the desulphurization of liquid fuel operating conditions.

Artificial intelligence tools have been invented to forecast the sulphur content of liquid fuel [12], and they are being used more frequently in a few energy sectors to track the adverse environmental and climatic effects of fossil fuels, which may then be used to mitigate such effects [19]. The reader can explore some publications on the successful application of artificial intelligence in the petroleum sector over a decade. A few of the applications include optimization and prediction of octane number loss while reducing olefin and sulphur contents in the gasoline refining process [20], prediction and modelling of petroleum reservoir characterization [21], forecasting of carbon dioxide corrosion in pipelines [22], leakage detection in gas and liquid fuel pipelines [23] and optimization and modelling of desulphurization process [[24], [25], [26]]. In addition, applying the fourth industrial revolution (4IR) technologies for desulphurization of liquid fuels will improve the global macro-economy, conserve energy, revolutionize petroleum refineries, and promote environmental sustainability [27].

Al-Jamimi et al. [12] reviewed the application of machine learning methods for optimization and modelling simulations of the desulphurization of refinery products. The study reported the relevant techniques involved in machine learning applications for the hydrodesulphurization of refinery products. It focused mainly on the supervised machine learning approach by considering parametric and non-parametric models to predict and optimize hydrodesulphurization. Nevertheless, hydrodesulphurization cannot remove aromatic refractory sulphur such as thiophene and its derivatives from liquid fuel during the catalytic reactions at high operating conditions, making the technique expensive and inadequate to produce cleaner fuel. Moreover, the study by Mguni et al. [28] employed multiple linear regression (MLR) and random forest (RFs) as machine learning tools to explore the complex adsorptive desulphurization process using zeolites as adsorbents. The study used data from the literature and related traditional modelling approaches with RF to examine adsorptive desulphurization. The MLR analysis was accompanied by caveat violations of linearity using a pairwise linear model, and the plot has outliers due to the introduction of bias and assumptions. For the RF model, the initial concentration of the fuel could not be controlled, and the partition coefficient was used to assess the adsorbent's performance, which is suitable for low concentrations. In addition, there are a few correlations that the study could not explain due to the limitation of using zeolites as adsorbent for desulphurization, and the study could not capture adequate data for micropore volume, mesopore volume, and pore size. The ANN predictive model's deep learning may have offered greater precision and predictive power than the RF model. Still, ANN's interpretability is complex, and RF was chosen due to its straightforward interpretability [28].

**Fig. 2:**
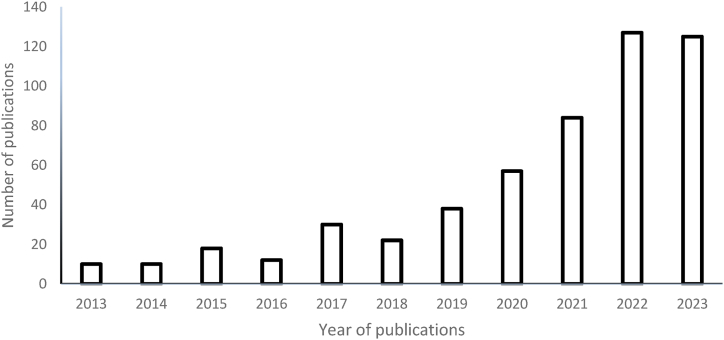
Scientific publications on optimization of the desulphurization process using artificial intelligence (data obtained from www.sciencedirect.com on August 22, 2023 using the keywords “desulphurization” and “machine learning”).

In addition, there are 533 publications with the keywords: “desulphurization” and “machine learning” from 2013 to 2023, as shown in Fig. 2 (data obtained from Science Direct on August 22, 2023), thereby signifying the goal of this study on the recent developments in the machine learning for desulphurization of liquid fuels. From the number of publications, it can be inferred that there is a need for more studies to be carried out on applying machine learning to the desulphurization of refinery products for environmental sustainability. Moreover, the challenges with incorporating 4IR technologies for desulphurizing liquid fuels and their future perspectives are elaborately highlighted in the next section to provide the reader with insightful information on the environmental sustainability of 4IR technologies for producing cleaner fuels.

## 3 Challenges and future perspectives of 4IR technologies for desulphurization of organic sulphur compounds in liquid fuels

Implementing the fourth industrial revolution (4IR) technologies in the petroleum industries has been perceived to boost productivity and mass production of refinery products. However, the full implementation of 4IR technologies for the desulphurization of liquid fuels ensures that some associated challenges threaten cleaner fuel production, and these challenges are amplified in most developing countries. The technologies are challenged by the large volume of liquid fuels to be desulphurized, and it is not cost-effective, specifically in the applications of nanotechnology and artificial intelligence techniques. Likewise, the instability in the efficiency of a fixed-bed adsorption column for the continuous desulphurization of liquid fuels using nanomaterial as an adsorbent is still a challenge [29,30]. Another challenge is the insufficient desulphurization process data and operation difficulties, which have hindered the application of 4IR technologies, especially artificial intelligence technology, to learn and solve specific problems associated with the desulphurization process [12].

Modern machine learning (ML) models have demonstrated considerable promise in bioenergy research, although they have not yet been fully implemented. The availability of data for creating a particular ML model is another significant obstacle to the application of ML technology in bioenergy systems [19]. Creating ML-based models requires expensive and time-consuming enormous volumes of training data. The black-box character of some ML algorithms, which restricts the interpretability of models and data, makes it challenging to utilize a mechanistic understanding of the desulphurization process, another barrier to applying ML models for desulphurization [19]. Therefore, more precise and specialized prediction algorithms are required to generate more comprehensive models that foresee a broader range of crucial aspects, including scientific data [31]. Since the results of other processes vary with different levels of complexity, a single ML approach is inadequate to forecast and analyse various data sets [32]. Incorporating several machine learning models or integrating them with other advanced techniques, including metaheuristic optimization models or complex statistical tools, has shown promise in the petroleum industry. Researchers have lately investigated and analysed hybrid models to represent various industrial processes. Although they require more computing labour, these hybrid models often outperform standard models in accurate prediction [33,34]. In order to produce cleaner fuel, there remains a research discrepancy in the use of ML systems in an integrated biorefinery that needs to be filled by developing innovative studies. The various ML models can significantly lower associated process costs, aid in better decision-making, and effectively assist with achieving the SDGs.

Nonetheless, the future perspectives of 4IR technologies are the growing demands to meet the global standard of sulphur content (≤10 ppm) in liquid fuels that have led to the invention of cost-effective, renewable, and efficient nanomaterials which can be used as adsorbents in the adsorptive desulphurization process. In addition, the incorporation of nanotechnology for the desulphurization of liquid fuels will lead to the selective adsorption of organo-sulphur and ultra-deep desulphurization of liquid fuels, thus producing cleaner fuels for further applications in automotive fuel cells and electric power generation [35]. Likewise, the removal efficiency of organic sulphur compounds from liquid fuels using nanomaterials as adsorbents can be simulated and modelled by applying artificial intelligence or machine learning techniques to predict and better understand desulphurization operations [12]. Hence, combining nanotechnology and artificial intelligence technology in desulphurization would yield outstanding results.

Similarly, the adequate prediction of the process operations by applying 4IR technologies to develop optimization models will enable petroleum industries to save operation costs, reduce process time and energy consumption. Moreover, 4IR technologies will help engineers detect and solve complex problems associated with liquid fuel desulphurization. In addition, the technologies can predict product yield and quality, and optimize the desulphurization process conditions to meet the global standards for liquid fuels with sulphur content ≤10 ppm, thereby increasing the market value of the products [36]. Also, applying 4IR technologies to the desulphurization process will ensure safety, adequate equipment maintenance, and reduced inefficient process models. Besides, the technologies will cause the existing industries to upgrade their process to boost productivity and embrace the new technological reality, producing cleaner fuels for transportation and power generation to drive socio-economic development [36]. The following sections give a detailed overview of the different techniques of desulphurizing liquid fuels to equip the reader with fundamental information that might help in understanding the other parts of this review.

## 4 Desulphurization of liquid fuels

Refinery products are made up of different chemicals and fuels for various applications, and one of the main activities for removing undesirable components, such as sulphur, from liquid fuels is desulphurization [26]. Liquid fuels (e.g., gasoline) are used chiefly as transportation fuels, and the presence of sulphur in these fuels has led to the discharge of sulphur oxide into the atmosphere, which is detrimental to human health and the environment [20]. Therefore, desulphurization is required to remove sulphur compounds from liquid fuels for environmental sustainability. Numerous variables, including sulphur removal rates, feed compositions, operating conditions, and catalyst activity, continue to impact the desulphurization process and affect the economy and environment [26]. The standard techniques for desulphurization of liquid fuels are as follows: hydrodesulphurization (HDS), extractive desulphurization (EDS), biodesulphurization (BDS), adsorptive desulphurization (ADS), and oxidative desulphurization (ODS). In addition, these desulphurization techniques have advantages and challenges/limitations, as summarized in Table 1, and the following subsections discussed optimizing desulphurization parameters by integrating artificial intelligence tools.

**Table 1:** Advantages and limitations/challenges of desulphurization techniques.

Desulphurization Technique	Advantages	Limitations/Challenges
Hydrodesulphurization (HDS)	Effective removal of aliphatic sulphur compounds, e.g., disulphide, thiols, and thioether	Unable to remove aromatic refractory sulphur, e.g., thiophene and its derivatives. It involves catalytic reactions at high operating conditions. It is an expensive technique.
Oxidative desulphurization (ODS)	Use suitable oxidants to convert the sulphur compounds to sulfones or sulfoxides at mild conditions. It is not energy-consuming, and no generation of harmful by-products	Further treatment, such as solvent extraction or any suitable method, is required. Selection of appropriate oxidants. Undesirable side reactions. Uneconomical technique.
Extractive desulphurization (EDS)	Selectively remove organic sulphur compounds at ambient conditions	Selection of suitable solvent to remove organic sulphur compounds.
Biodesulphurization (BDS)	Removal of sulphur from organic sulphur compounds via enzymatic reactions. The technique is environmentally friendly, and there is no generation of hazardous by-products. Immobile enzymes can be reused many times without a further separation process.	Separating microbial cells after the complete reaction is complex. Cell immobilization can lead to diffusional restrictions. Maintaining the optimal conditions for the development of microbes is complex. Enzyme deactivation might occur during the process.
Adsorptive desulphurization (ADS)	It is cost-effective and operated at ambient conditions. It is eco-friendly, and hazardous by-products are not formed. Regeneration and reusability of adsorbents are easy.	The adsorbent determines the technique's efficiency, and adsorbent properties influence the adsorption performance. Selectivity and affinity for aromatic sulphur organic compounds in liquid fuels vary with adsorbent.

### 4.1 Optimization of desulphurization process conditions using artificial intelligence tools

**Fig. 3:**
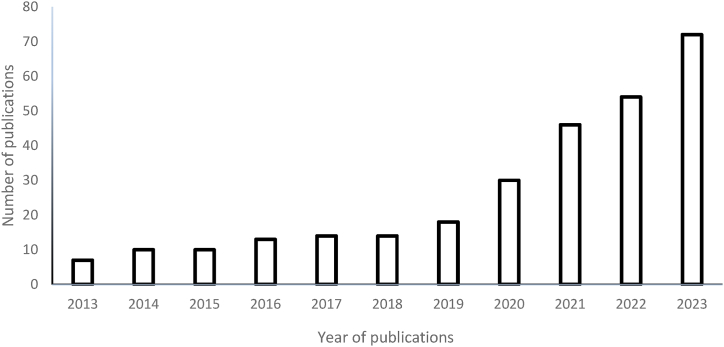
Scientific publications on optimization of the desulphurization process using artificial intelligence (data obtained from www.sciencedirect.com on August 11, 2023 using the keywords “optimization,” “desulphurization,” and “artificial intelligence”).

The concerns about the potential depletion of fossil fuels and their environmental damage have led to international legislation to reduce their dangers and promote the development of cleaner fuel alternatives [19]. It is critical to model and improve the adsorptive desulphurization process to promote the accomplishment of SDG 7 (cleaner energy) and ensure the incorporation of fossil fuels as lasting clean energy sources [19]. Various researchers have used different adsorbents for the adsorptive desulphurization of liquid fuel, as summarized in Table 2. However, the conventional techniques of modelling and optimizing the chemical process are used in refining. As a result of the diversity of equipment and nonlinear operating parameters, it is necessary to optimize the conditions and experimental parameters, which is costly and time-consuming [20]. The conventional methods necessitate a significant consumption of chemicals and resources, which are expensive in terms of cost, effect on the environment, and time required. Thus, using the data-generating strategy is advised for minimizing the number of trials and increasing output. Statistical models that relate to the response of the variables tested can be created using the data gathered through artificial intelligence techniques. Therefore, using artificial intelligence, quick and easy parameter optimization is exceptionally preferred in desulphurization [17]. Artificial intelligence, also called machine learning, has various applications in different approaches. However, modelling challenges in adsorptive desulphurization arise from identifying necessary operating conditions [28]. Therefore, recent studies have used and contrasted artificial intelligence algorithms to address these issues. The number of publications with the keywords: “optimization”, “desulphurization”, and “artificial intelligence” for over a decade (data obtained from Science Direct on August 11, 2023) is presented in Fig. 3. The publications gradually increased from 7 in 2013 to 72 in 2023, with a progressing research interest related to the keywords.

**Table 2:** Summary of adsorptive desulphurization of liquid fuel.

Adsorbent	Target sulphur compound(s)	Operating conditions	Adsorption capacity or % sulphur removal	Reference
Activated carbon manganese oxide	Thiophene, Benzothiophene, Dibenzothiophene	50 ppm, 25 °C, 0.5 g, 60 min	4.5 mg/g 5.7 mg/g 11.4 mg/g	[37]
Ceria nanorods	Thiophene	200 ppm, 25 °C, 0.5 g	2.5 mg/g	[38]
Ni/Cu-carbon nanofiber	Thiophene	35 ppm, 30 °C, 1 g, 130 min	0.5 mg/g	[39]
Modified clays	Dibenzothiophene, 4,6-dibenzothiophene	25 ppm, 25 °C, 25 mg	11.3 mg/g 4.7 mg/g	[40]
Pomegranate leaf powder	Dibenzothiophene	1000 ppm, 30 °C, 0.2 g, 3 h	70.55 %	[5]
Ni–Metal-organic framework	Thiophene	102 ppm, 30 °C, 100 mg, 3 h	4.74 mg/g	[41]
CeHY zeolite	Thiophene, 2-methyl thiophene, Tetrahydrothiophene Benzothiophene	300 ppm, 30 °C, 1.0 g, 5 h,	9.5 mg/g 28.6 mg/g 31.2 mg/g 10.6 mg/g	[42]
Neem Leaf Powder	Dibenzothiophene	1000 ppm, 30 °C, 0.8 g, 60 min.	65.78 %	[43]
Graphene Nanoplatelets	Thiophene, 2-methyl thiophene, Dibenzothiophene	1500 ppm, 25 °C, 3 g, 100 min.	117.21 mg/g	[44]
Metal-organic framework	Dibenzothiophene	800 ppm, 25 °C, 10 mg, 4 h	710 mg/g	[45]
Zn and Mn-loaded activated carbon	Dibenzothiophene	200 ppm, 30 °C, 0.15 g, 2 h	95.7 %	[46]
NiO/ZnO–TiO_2_	Thiophene	300 ppm, 340 °C, 45 mg, 2 h	98.3 %	[47]
Organoclays	Dibenzothiophene	1000 ppm, 45 °C, 0.5 g, 60 min.	70.8 mg/g	[48]
Metal-organic frameworks	Dibenzothiophene	1000 ppm, 30 °C, 5 h	112 mg/g	[49]
Cu-carbon nanofiber	Thiophene	2000 ppm, 30 °C, 20 g/L, 4 h	103.4 mg/g	[50]
Modified Metal-organic frameworks	Dibenzothiophene	1000 ppm, 25 °C, 0.2 g, 24 h	94.55 %	[1]
Nitrogen modified graphene	Thiophene, Benzothiophene, Dibenzothiophene	300 ppm, 70 °C, 0.1 g, 1 h	97.3 % 92.8 % 88.4 %	[51]

Furthermore, the optimization of a flue gas desulphurization system was explored by Liu et al. [24] using mined historical data, and the data were analysed using principal component analysis and enhanced fuzzy *C*-means. Still, the approach employed by the study could not provide the optimal operating conditions for the desulphurization process. Another study by Makomere et al. [52] applied RSM and ANN to model and optimize sulphur removal at low temperatures and assessed a flue gas desulphurization performance. The study used the central composite design of RSM to design the experiment using time, temperature, sulphur dioxide inlet concentration, diatomite to Ca(OH)_2_ ratio, and sulfation efficiency as dependent variables, with desulphurization efficiency as the response variable. The ANN model was developed using a 5-8-2 layout structure mapped with the Levenberg-Marquardt algorithm to assess the desulphurization performance. They observed that RSM and ANN models gave desirable and accurate predictions; however, underfitting and overfitting inefficiencies, as well as reduced hidden layers, influenced the performance of the ANN model. Nevertheless, adopting big data and artificial intelligence technologies with outstanding goodness of fit in desulphurizing liquid fuels make it possible to collect, process, and analyse extensive data by intelligent algorithms for actual prediction and optimization of the desulphurization processes [53].

Al-Jamimi et al. [17] also examined the modelling and optimization of the hydrodesulphurization process using an intelligent technique. The study applied a machine learning approach by using a support vector machine (SVM) model coupled with a genetic algorithm (GA) to enhance the optimization and predictive capability of the SVM model. They developed the hybrid model to predict sulphur content in the hydrodesulphurization process with better configuration to optimize the process. However, they reported that the SVM's prediction ability heavily depends on the precise composition of its parameters, including the insensitive loss function, regularization parameters, and radial basis function kernel. Due to the incorrect selection of SVM parameters, the effects of model cross-training continue to be significant factors [17]. In a similar study by Al-Jamimi et al. [26], a hybrid model (SVM and GA) was employed to optimize the hydrodesulphurization process based on initial sulphur content, inlet temperature, catalyst dosage, and pressure. The SVM was chosen because of its scalability with high dimensional data and relative simplicity of learning. The main drawbacks of the studies earlier examined are the choice of technique for desulphurization and the artificial intelligence tool utilized for desulphurization optimization and prediction; hence, one of the goals of this review is to explore other artificial intelligence tools with improved performance for adsorptive desulphurization process. Some examples of artificial intelligence tools that have been applied to optimize, predict, and model desulphurization processes are presented in Table 3.

**Table 3:** Examples of how artificial intelligence tools have been used for predicting and modelling industrial processes.

Artificial Intelligence Tool	Industrial Process	Significant Observation	Reference
GA–SVM	Molecular hydrodesulphurization using a catalyst	The hybrid model corresponded with experimental data	[17]
ANN	Optimization and modelling of a flue gas desulphurization process	Improved prediction accuracy of the outlet concentration and optimized operating conditions	[54]
CMAC – Neural Network and GA	Optimization and prediction of a desulphurization system	The outcome demonstrated that under the same limitations, GA performed better with reduced output errors	[55]
Principal component analysis and fuzzy *C*-means	Optimization of the desulphurization process	Optimized operating parameters, with upgraded desulphurization efficiency and economic benefits	[24]
SFS and DE	Prediction of octane number loss in gasoline	The model showed the least mean square error compared to SVM and RF	[20]
RF and MLR	Adsorptive desulphurization using zeolite	RF model predicted better than the MLR model	[28]
SVM and GA	Hydrodesulphurization (HDS) process in petroleum refinery	The models adequately optimized HDS yield with high accuracy	[26]
Neural Network	Prediction of impurity removal from atmospheric residue desulphurization process	Adequate predictions were produced by the trained models and met the chemical stability requirement.	[56]
ANN and RSM	Modelling of flue gas desulphurization system	ANN model gave better accuracy than the RSM model	[52]
Grey–box with GA, Metropolis-Hasting and NN	Desulphurization of hot metal in steel production	Efficient algorithm with rapid computation time and precision.	[57]
ANN and semi-empirical regression	Temperature profile prediction of Gasoil HDS process in petroleum refinery	ANN model performs better than regression models when predicting reactor bed temperature.	[58]

ANFIS – Adaptive Neuro-Fuzzy Interference Systems, ANN – Artificial Neural Network, RF – Random Forest, MLR – Multiple Linear Regression, SVM – Support Vector Machine, GA – Genetic Algorithm, SFS – Sequence Forward Search, DE – Differential Evolution, CMAC – Cerebellar model articulation controller, NN –Neural Network.

### 4.2 Incorporation of Adaptive Neuro-Fuzzy Interference Systems and artificial neural networks in desulphurization process

**Fig. 4(a):**
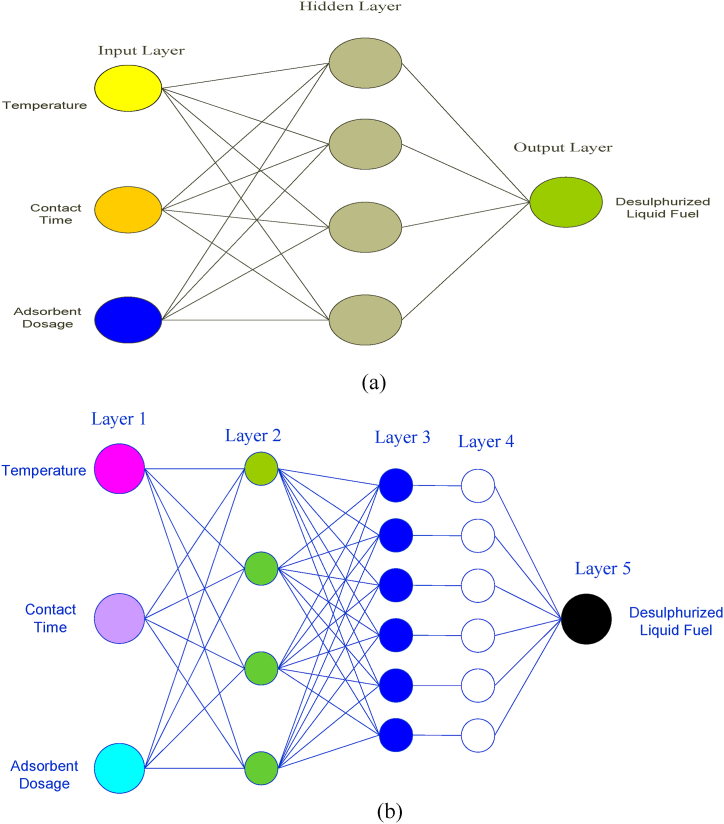
Artificial Neural Network (ANN) structure for desulphurization of liquid fuel Fig. 4(b) Adaptive Neuro-Fuzzy Interference System (ANFIS) structure for desulphurization of liquid fuel.

Adaptive neuro-fuzzy interference systems (ANFIS) and artificial neural networks (ANN) are artificial intelligence techniques for predicting and modelling industrial processes. The artificial neural network (ANN) is an intelligent mathematical data processing tool that uses an algorithm inspired by a biological neural system called neurons to learn, predict, and optimize process data without introducing assumptions about the nature and interrelations of the data [16,59]. The neurons are divided into three layers, as presented in Fig. 4(a): the input layer, which receives the input information; the hidden layer, where mathematical operations are performed; and the information obtained will be the input for the last layer, and the output layer [60]. ANN integrates collected input data, combines the data by performing nonlinear multivariate operations, and generates the outputs without creating any assumption about the nature and interrelations of the input data [61]. Unlike ANN, ANFIS is constituted by five networks or multilayers joining directly to one another, as shown in Fig. 4(b), and the order of arrangement of the layers for their functions includes fuzzification, multiplication, normalization, defuzzification, and summation [16].

Conversely, the adaptive neuro-fuzzy interference system (ANFIS) algorithm combines fuzzy logic's reasoning and ANN's learning ability. The ANFIS model applies membership functions, a back-propagation technique based on artificial intelligence and IF-THEN rules produced according to its knowledge [29,60]. ANFIS depends on dependent and independent variables to learn membership functions, understand the system behaviour during training, and achieve the IF-THEN rules [61]. Therefore, it was reported that the better performance of ANN predictability over ANFIS could be because of overtraining data and fuzzy rule-based complexity observed in the ANFIS model [62]. Although neural networks have excellent fitting performance, they are prone to overfitting, mainly when there is a restricted sample size. Cui et al. [20] suggested that the decision tree method can be applied to overcome the drawbacks of ANN. Compared with the neural network model, the decision tree model is advantageous for optimizing chemical processes due to the model's ease of implementation, resistance to overfitting, and superior interpretability. Additionally, due to concerns that using ANN would result in flattening or not exploiting all process conditions and processes, scientists regularly compare ANN with algorithms like random forests, adaptive neuro-fuzzy inference systems, and support vectors [63,64].

Artificial Neural Networks (ANNs) are utilized in various situations where nonlinear RSM might not be the best choice because of its adaptability. ANNs can get around the drawbacks of the traditional method, eliminate the need to create a model, simplify things, and give the best possible output parameters [61,62]. Even though ANN is frequently used in real-world systems, there are still challenges with long learning curves, performance fluctuations, and managing ambiguous data. Adaptive Neuro-Fuzzy Inference System (ANFIS) has begun to replace ANN for modelling intricate industrial systems to overcome ANN's shortcomings. ANFIS offers a straightforward architecture with the ability to adapt the challenging translation of human brains to fuzzy systems, capable of handling the complexity and unpredictability of real-world problems, and suitable logic capacity in addition to resolving the issues with ANN [60,63].

On the other hand, the inability to quickly discover a membership function, high computing expenses, extreme susceptibility to input factors and basic fuzzy rules, and difficulty interpreting precision choices are the critical drawbacks of ANFIS [65]. Thus, combining two or more artificial intelligence tools is advocated to solve the challenges with a single artificial intelligence tool. It is worthy of note that beyond the potential incorporation of artificial intelligence tools into the desulphurization process, these tools have been applied to predict and optimize the valorization of waste biomass. The following section provides an overview of the application of artificial intelligence tools to valorise waste biomass for environmental sustainability.

## 5 Application of artificial intelligence in waste valorization to attain sustainable development goals (SDGs)

Traditional modelling techniques like the Taguchi method, one-variable-at-a-time system, and genetic algorithms may be beneficial when dealing with complex experimental data [19]. These methods are ineffective for accurate model approximation to optimize experimental conditions, leading to subpar model creation. As a result, artificial intelligence systems can aid in locating the most significant resources that are presently accessible and increasing feedback accessibility for long-term use [19]. In the past ten years, there has been a noticeable change from physical modelling to data-driven modelling in developing modern lignocellulosic biorefineries, which has increased interest in using machine learning or artificial intelligence technologies [66]. Researchers in various scientific sectors, including the bioenergy industry, have greatly encouraged the application of artificial intelligence (AI). AI enables machines to mimic some elements of human brain function using diverse computer science approaches like heuristic algorithms, machine learning, and fuzzy logic [67,68]. Artificial intelligence (AI) technology, known as “machine learning” or “deep learning”, uses mathematical techniques on data obtained on its own to increase performance and accuracy [69]. AI provides a choice of advanced bioenergy production systems for efficient natural resource utilization and environmental awareness [70].

Integrating artificial intelligence (ML) and traditional chemical characterization approaches may considerably improve the advancement of highly valuable processing of lignocellulosic biomass (LCB) [65]. To analyse and optimize the initial results of LCB indicators, identify the primary influencing elements, and boost the efficacy of resolving real-world research bottlenecks, combining ML and conventional analytical characterization methods is more realistic and practical than utilizing them alone [71]. In general, different mathematical models and ML implementations can effectively optimize the parameters of the data obtained by conventional physicochemical characterization techniques (for example, thermogravimetric analysis, Fourier transform infrared spectrometry and nuclear magnetic resonance) [72]. To anticipate the impact of chemical treatment on lignin, Castro et al. [72] evaluated ML methods such as artificial neural network (ANN) and support vector machine (SVM) in addition to conventional thermogravimetric analyses. The effect of chemical treatment and temperature on lignin decomposition causes the prediction accuracy of ANN and SVM algorithms to decrease. Moreover, Velidandi et al. [65] explored the application of machine learning procedures to the characterization, pretreatment, product yield and quality, bioconversion process, and valorization of waste biomass. However, their review did not capture the application of ML algorithms for extracting nanocellulose crystals and green pretreatment methods of lignocellulosic biomass in the biorefinery process. As highlighted in this current review, one of the critical aspects of study for waste valorization is emerging machine learning algorithms for green isolation techniques and optimizing the process parameters to promote the nanocellulose crystals’ quality, economic viability, and environmental sustainability.

**Fig. 5:**
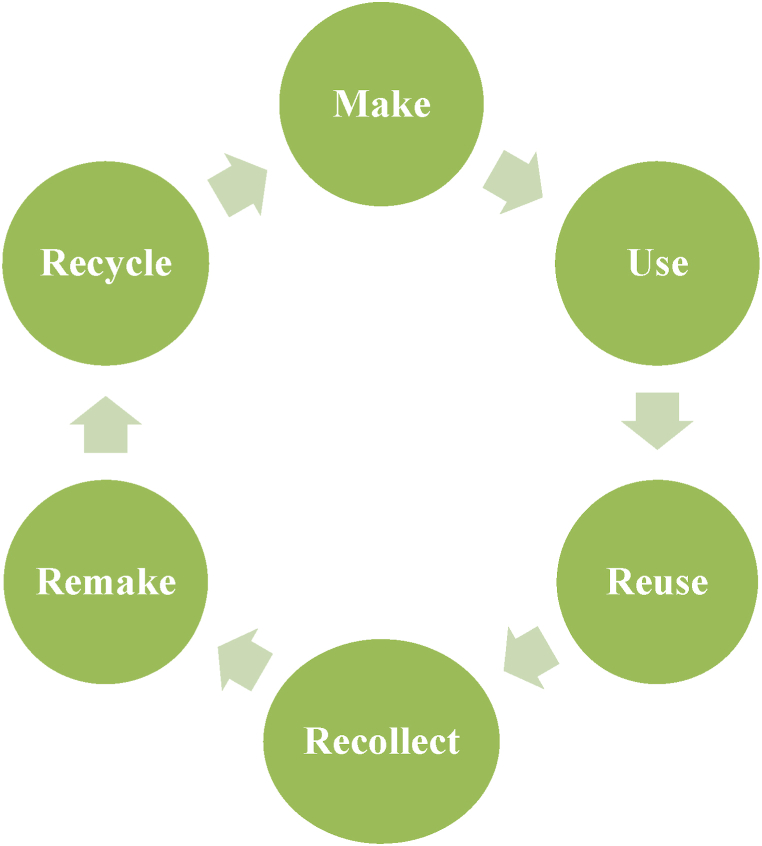
Waste valorization for circular economy and environmental sustainability.

Additionally, Chen et al. [73] recommended that due to the high cost of pretreatment necessary for the valorization of lignocellulosic biomass, it is crucial to develop sustainable green techniques and optimize the process conditions for enhanced efficiency. It is worth stating that several lignocellulosic biomass wastes have been successfully transformed into valuable products (e.g., nanocellulose crystals). Furthermore, the actualization of the circular economy, as shown in Fig. 5, via waste valorization is enhanced when green isolation techniques are employed [74,75], and the methods can be improved by using artificial intelligence tools to model and optimize the fractionalization of waste biomass into value-added products. The application of artificial intelligence tools for modelling and optimizing green isolation techniques to achieve waste valorization is highlighted in the following section of this review.

## 6 Optimization and modelling of green synthesis strategies for fractionalization of waste biomass

The operating conditions for fractionating waste biomass must be enhanced, which may be done by modelling the operating parameters and choosing the best process parameters to meet the market's demands. Various models, including response surface methodology (RSM), artificial neural networks (ANN), and genetic algorithms (GA), have been created and applied in biorefinery operations, as was covered in the previous section. However, conventional optimization tools were formerly used to develop mathematical models for valorizing waste biomass [19]. The development of artificial intelligence algorithms is quickly replacing conventional modelling tools to save time, cost, and energy. Traditional stoichiometric procedures lack the durability, significant learning capability, and risk tolerance of artificial intelligence, which makes artificial intelligence a more realistic and convincing scientific approach [76]. More specifically, artificial intelligence is used in biorefinery systems for noise filtering, supply chain modelling, recognizing patterns, end-use system performance, and optimization [68].

Artificial neural networks (ANN), one of the standard artificial intelligence tools, have been extensively employed as predictive tools in various study fields due to their learning capacity, even with little experimental data. ANN is also used for linear and nonlinear systems [77]. Unlike RSM, ANN does not require a prior fitting method to estimate nonlinear functions like polynomial and quadratic functions. The efficiency of the ANN depends on how uniformly the data are statistically distributed, and RSM can only match quadratic functions and require fitting procedures [78]. The study by Wijaya et al. [79] applied RSM to optimize the isolation of nanocellulose crystals from bamboo shoots, and the fitting of independent parameters toward the response variable influenced the statistical significance of the model. However, Selvakumar et al. [80] investigated the optimal parameters for acid hydrolysis of lignocellulosic waste. They used RSM and ANN modelling to focus on the temperature, pH, time, and agitation as process variables. The study generated 30 trials using the central composite design of RSM with six centre points to design the experiment and determine how the four process factors affected the bioconversion of lignocellulosic waste. Also, the ANN model was developed using a 1-8-1 structure, and the network was trained by applying a variable learning rate back algorithm with the four process variables. They claimed that the ANN model was preferable to the RSM since there was a strong connection between experimental and predicted data. However, acid hydrolysis used in the study is not eco-friendly due to the generation of hazardous by-products.

Additionally, the study by Rashid et al. [81] optimized the operating parameters for isolating lignin from waste biomass using ANN and RSM. Before employing RSM to optimize, the Box-Behnken design was used to determine the effects of various operational parameters on the efficiency of lignin extraction, including extraction time, temperature, and biomass loading. Also, the ANN modelling involved a feed-forward neural network with multilayer perception in predicting lignin extraction, and Levenberg-Marquardt (LM) was adopted for training the data set. The performance of the ANN and RSM models were compared, and the ANN model performed better than the RSM model. Although the ANN structure was designed using up to three hidden layers and neurons varying from five to twenty to reduce prediction error, the model showed few errors with the predicted data.

Furthermore, Wang et al. [82] applied three machine learning methods, namely ANN, random forest (RF), and decision regression, to predict and analyse the extraction of nanocellulose crystals from forty different cellulose feedstocks based on published data from the literature. The focus of the study was the crystallinity prediction of the extracted nanocellulose crystals, and the crystallinity prediction accuracy of the three machine learning methods was lower than the yield prediction. This observation was attributed to the isolation technique and insufficient data to train the models. Likewise, the method and equipment used influenced the measurement accuracy of crystallinity. The study could not optimize the isolation conditions due to uncertainty regarding the cellulose feedstock, isolation technique, pretreatment approach, and required chemicals. Furthermore, the green isolation strategies for extracting nanocellulose crystals from different waste biomass have been successfully achieved mainly by applying liquid hot water (hydrothermal) pretreatment and ionic liquids pretreatment, as depicted in Table 4. In addition, for the optimization of lignocellulosic biomass pretreatment, different input variables are considered depending on the goal of the pretreatment process, and the optimization of the two main green isolation strategies is discussed in the succeeding subsections.

**Table 4:** Extraction of nanocellulose crystal from waste biomass via green synthesis.

Waste biomass	Green synthesis technique and operating conditions	% Yield	Crystallinity (%)	Reference
Corn stover	Ionic liquid 140 °C, 3 h, 50 % w/w	88		[83]
Poplar (*Populus trichocarpa*)	Liquid hot water 180 °C, 70 min, 5 % w/w	62.00	58.80	[84]
Sugarcane straw	Liquid hot water 120 °C, 7 h, 3 % w/v	86.50		[85]
Wheat Bran	Ionic liquid 150 °C, 40 min, 5 % w/v	83		[86]
Corncob	Liquid hot water 190 °C, 30 min, 10 % w/w	78.34	50	[87]
Cotton linter	Ionic liquid 100 °C, 2 h, 10 % w/w	33.1	69.6	[88]
Napiergrass (*Pennisetum purpureum*)	Liquid hot water 200 °C, 15 min, 11 % w/v	73.00		[89]
Rubber wood Maize husk	Ionic liquid 125 °C, 1 h, 10 % w/w		66.9 59.3	[90]
Green pepper waste	Liquid hot water 150 °C, 40 min, 10 % w/v	55		[91]
Rice straw	Ionic liquid 120 °C, 5 h, 5 % w/w	64.90	62.20	[92]
Coconut waste	Ionic liquid 170 °C, 45 min, 10 % w/w	89		[93]
Corncob	Liquid hot water 200 °C, 60 min, 10 % w/w	55.5	57.3	[94]

### 6.1 Optimization and modelling of liquid hot water pretreatment

Liquid Hot Water (LHW) is known as autohydrolysis for the pretreatment of waste biomass to enable enzymatic solubilizing of hemicellulose. It removes up to 80 % of hemicellulose from different lignocellulosic materials [95]. LHW enhances the saccharification process of polysaccharides, especially cellulose, by significantly reducing the recalcitrance of the lignocellulosic biomass's cell walls and the pretreatment technique is regarded as a sustainable approach for bioenergy generation [96]. The technique has been applied for the pretreatment of various biomass, including corncob [87,94], corn stover [97], macroalgae [98], green pepper waste [91], Hybrid *Pennisetum* [99], wheat straw [73,100], walnut shells [101], bamboo [102] and sugarcane straw [85]. Therefore, examining the optimal pretreatment operating conditions is significant to achieve the highest cellulose recovery from lignocellulosic biomass using liquid hot water pretreatment. In a recent study, Bekker et al. [103] evaluated the optimization of liquid hot water pretreatment conditions for the pretreatment of clover-grass press cake, and they used pretreatment time and temperature as the operating variables. The optimization was carried out based on the findings of a one-way ANOVA, examined using Tukey tests.

Furthermore, Kang et al. [99] optimized the process conditions (time, temperature, and ratio of liquid to solid) of liquid hot water pretreatment on Hybrid *Pennisetum* using a central composite design of response surface methodology. At the optimal pretreatment conditions, the biodigestibility of Hybrid *Pennisetum*, the process energy efficiency and the biomethane yields were enhanced. Varongchayakul et al. [104] also applied response surface methodology (RSM) to optimize liquid water pretreatment on cassava pulp. A central composite design was used to model the pretreatment process, and reaction time and temperature were the main factors considered in the pretreatment optimization. The statistical evaluation showed pretreatment temperature as the most significant factor influencing the degradation of hemicellulose, and experiment validation was required to predict the optimum pretreatment conditions appropriately.

In contrast, in another recent study by Forsan et al. [105], acid hydrolysis and autohydrolysis processes were optimized using RSM, and the effects of the two hydrolysis methods were compared to treat sugarcane leaves. A central composite design was used to optimize the liquid hot water (autohydrolysis) treatment, and the treatment circumstances (treatment duration and temperature) were divided into three stages. The model for the autohydrolysis presented reaction temperature and time as the significant factors influencing the process, and the model was used to determine the treatment conditions where undesirable by-products were generated. Likewise, they observed that temperature harmed the acid hydrolysis process because elevated temperature, reduced hydrolysis time, and low acid concentration promoted the yield. On the other hand, a decrease in temperature required higher time and acid concentration to obtain a similar range of output, making the process unsustainable and environmentally unfriendly.

Similarly, Olawuni et al. [94] investigated the optimization of liquid hot water on corncobs using a central composite design of response surface methodology, and the process was modelled based on temperature, solid loading rate, and reaction time as the pretreatment variables. They noticed a deviation between the predicted and actual yield due to outliers that could be attributed to minor errors; thus, the developed RSM model needed perfection. In another study, Varongchayakul et al. [106] evaluated liquid hot water pretreatment of atratum and ruzi grasses before anaerobic digestion. Likewise, a central composite design modelling of response surface methodology was employed to assess the pretreatment process optimization. They presented that the LHW pretreatment improved methane yield and biodegradability of the substrates during anaerobic digestion. Most parameters considered during optimizing lignocellulosic pretreatment are based on the solid residue or liquid after pretreatment, as opposed to high cellulose content and low hemicellulose and lignin. The purpose of their study for optimizing LHW pretreatment conditions (temperature and time) was to enhance biodegradation and methane yield, and there was a variation between the coefficient of determination values for the actual and adjusted data.

Chen et al. [73] also examined the optimization of subcritical water pretreatment of wheat straw conversion using CCD of RSM. During the optimization of subcritical water pretreatment, it was shown that the breakdown of the resistant structure of wheat straw and cellulose degradation was in equilibrium. However, the challenge with the method was the crucial need to find the balance between wheat straw and cellulose degradation during the pretreatment and preserve the point for optimum yield. It was reported that the main reason for pretreatment is to improve enzymatic hydrolysis, and a higher R^2^ value was obtained for pretreatment only than combined enzymatic and pretreatment evaluation. The optimization of LHW has widely been done using RSM, hence the importance of this subsection and the potential application of artificial intelligence to optimize the LHW pretreatment variables. Therefore, the application of artificial intelligence to predict and optimize the LHW pretreatment conditions is crucial for treating different feedstocks and enhancing the hydrolysis process during the transformation of lignocellulosic biomass to nanocellulose crystals and other valuable products.

### 6.2 Optimization and modelling of ionic liquids pretreatment

Anions and cations combine to form a class of salts known as ionic liquids (ILs), which are liquid at room temperature. Ionic liquids are used more frequently in the pretreatment of lignocellulosic biomass because of their unique physicochemical characteristics, which include their high solution ability for the dissolution and hydration of lignin and carbohydrates, their adequate chemical and thermal reliability and their low vapour pressure [107,108]. The main benefit of utilizing ILs for pretreatment is the low loss in solvent recovery, renewability, and reusability. However, the drawbacks of this technique are the need for many expensive ILs, the need to recover the hemicellulose and lignin following cellulose isolation, and the sticky and tough-to-handle solutions that develop during the procedure [109]. Nonetheless, Mesa et al. [110] suggested the dilution of ionic liquids with water to prevent this problem and promote this environmentally friendly pretreatment technique. Likewise, Babicka et al. [111] stated that cations, anions, source of cellulose, temperature, and extraction time are the main factors influencing the ionic liquid-based fractionation of lignocellulosic biomass. Additionally, Babicka et al. [112] reported that nanocellulose could be extracted and recovered after ionic liquid pretreatment of microcrystalline celluloses, making the technique more amenable as a renewable chemical raw material for other applications, thereby leading to reduced costs of operation. In addition, to adequately optimize the ionic liquid pretreatment conditions and predict cellulose recovery, artificial intelligence (machine learning) can be applied to model the essential input variables, design experiments based on the model interpretation, and better understand the process.

Besides, Phromphithak and colleagues [113] studied the optimization of ionic liquid pretreatment of lignocellulosic biomass using three different machine learning algorithms, namely support vector machines (SVM), random forest (RF), and gradient boosting (GB). The algorithms were applied to predict the production of cellulose-rich materials and solid recovery from the lignocellulosic biomass. They used 23 data sets and divided them as 75 % for training and 25 % for testing to optimize the model parameters. The RF algorithm gave the highest prediction accuracy compared to other algorithms due to its advantage of handling extensive data and combining several decision tree algorithms. It was noticed that high temperature and reaction time harmed the production of cellulose-rich materials and solid recovery. Hence, a two-way partial dependence plot was recommended to optimize the process and new ionic liquid pretreatment.

On the other hand, in the study by Smuga-Kogut et al. [64], the five distinct ionic liquid pretreatments of mugwort and hemp biomass were improved using random forest (RF) and artificial neural network (ANN) algorithms. The outcome demonstrated enhanced efficacy of both RF and ANN models with coefficient of determination (R^2^) values of 0.95 and 0.9, respectively. In addition, the study discovered that the random forest algorithm performed better with a substantial prediction accuracy than the ANN algorithm; hence, the R^2^ value is influenced by the applied model. Due to the different biomass wastes used in the study and process complexity, it was tough to obtain accurate results applying only a single algorithm. Therefore, a hybrid modelling strategy will increase the accuracy of the results and reduce the requirement for time-consuming and expensive research on IL-based treatments.

Similarly, 1-ethyl-3-methylimidazolium acetate pretreatment was investigated by Saha et al. [114] on sugarcane bagasse. A central composite design (CCD) of response surface methodology (RSM) was used to optimize the pretreatment conditions (time, temperature, and the ratio of ionic liquid to bagasse), and an empirical model was created to look at the interactive impact of the isolation conditions on yield. The modelling revealed that extended pretreatment time, increased temperature, and high loading rate favoured the yield response. However, these conditions are not cost-effective and could lead to cellulose degradation and production of inhibitory by-products. In a recent study, Mesa et al. [110] investigated the optimization of 1-butyl-3-methylimidazolium chloride (BmimCl) pretreatment of sugarcane bagasse using a central composite rotatable design based on the solid loading rate, time and temperature as process variables. They observed reduced sugar loss after the pretreatment and improved digestibility after the enzymatic hydrolysis at the optimum conditions. Likewise, Nurdin et al. [115] studied the optimization of triethylammonium hydrogen sulphate (IL [TEA][HSO_4_]) as an ionic liquid for the pretreatment of empty oil palm fruit bunches. The study optimized pretreatment temperature and ionic liquid composition as the two factors influencing the transformation of the empty oil palm fruit bunches. However, the study could not achieve improved ionic liquid recovery at the optimum condition, and the impact of pretreatment time was not investigated.

Furthermore, Poy et al. [92] examined the pretreatment of rice straw using 1-ethyl-3-methylimidazolium acetate and observed that the ionic liquid pretreatment disrupted the complex lignocellulosic matrix of the rice husk. Also, they applied response surface methodology (RSM) to optimize the pretreatment parameters and maximize delignification and enzymatic hydrolysis yield. The RSM model had limited performance in predicting the best pretreatment parameters. In a similar study, Araya-Farias and co-workers [86] applied 1-ethyl-3-methylimidazolium acetate for the pretreatment of wheat bran, and they optimized the pretreatment conditions (time, temperature, loading rate and concentration of ionic liquid in water) using partial least square and second order design. The optimization results showed that the partial least square quadratic model could be improved by adequately fitting the independent parameters using the second-order model. Therefore, this review strongly recommends intelligent modelling using artificial intelligence tools to overcome this drawback. In addition, the effect of cationic imidazolium ionic liquids on the isolation of cellulose nanocrystals was studied by Grząbka-Zasadzińska et al. [116], and the potential application of nanocellulose crystals as green adsorbent for efficient desulphurization of liquid fuels was highlighted in the following section.

## 7 Application of nanocellulose crystal (green adsorbent) for adsorptive desulphurization of liquid fuels

**Fig. 6:**
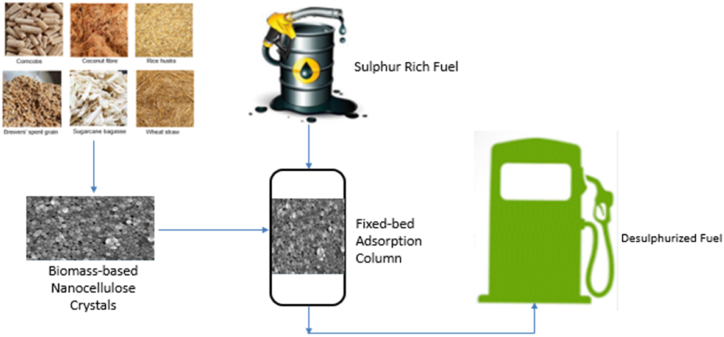
Application of biomass-based nanocellulose crystals for adsorptive desulphurization.

The recent applications of nanocellulose crystals for water and wastewater treatment are responsible for their potential application in the adsorptive desulphurization of liquid fuels due to their cost-effectiveness, adsorption efficiency, large surface area, mild operating conditions, and mechanical and thermal stability. Additionally, it is worth knowing that there are few publications on the utilization of green adsorbents extracted from waste biomass to remove sulphur organic compounds (e.g., dibenzothiophene and its derivatives) from liquid fuel, such as pomegranate leaf powder [5], palm kernel shell activated carbon [117], and neem leaves [43], as well as functionalized activated carbon from corncob [46]. Some waste biomass-derived adsorbents for adsorptive desulphurization are represented in Fig. 6. A more significant proportion of sulphur organic compound was successfully removed according to the adsorption capability of the adsorbent with minimal adsorbent dosage, and the surface functionality was significantly responsible for the adsorption capacity of the green adsorbents. Furthermore, Li and co-workers [118] reviewed the application of nanocellulose materials in the oil and gas industry as an environmentally friendly and sustainable approach to enhance the development and productivity in the industry. The review focused on the three main sectors of the oil and gas industry (upstream, midstream, and downstream sectors). In the downstream sector, the study explored the application of nanocellulose materials in the desulphurization of natural gas and crude oil using hydrodesulphurization and oxidative desulphurization techniques.

Similarly, the study by Zhang et al. [119] examined the performance of mesoporous alumina and NiMo catalysts using synthesized nanocellulose fibre as a template for removing dibenzothiophene via the hydrodesulphurization process. The result of the study revealed that the incorporation of nanocellulose fibre as a template in the mesoporous alumina and NiMo catalysts made it perform six times better than when it was not incorporated. However, the hydrodesulphurization process was carried out at an elevated temperature and pressure, making it expensive. Still, the mesoporous alumina and NiMo catalysts considered in the study were not green materials. Therefore, this review explored using biomass-derived materials as green adsorbents in adsorptive desulphurization as an alternative to the hydrodesulphurization technique. Based on the successful studies by different researchers on the application of green adsorbents for adsorptive desulphurization, there is a need for further research on the adsorption mechanism of nanocellulose crystals (green adsorbents) isolated from waste biomass for the adsorptive desulphurization of liquid fuels. The adsorption mechanism and surface chemistry influence the adsorbent's performance for effective desulphurization, and they are briefly discussed in the following sub-sections.

### 7.1 Adsorption mechanism of nanocellulose crystal for selective desulphurization of gasoline

**Fig. 7:**
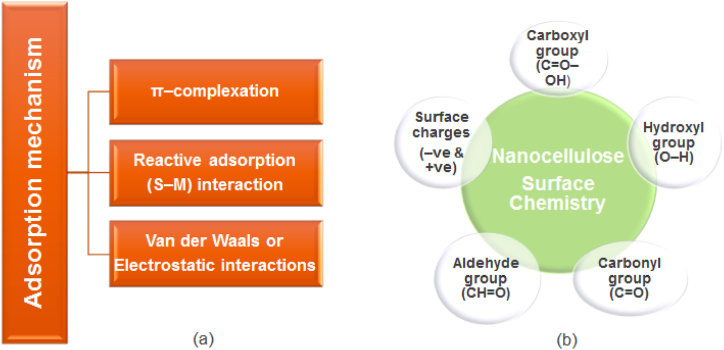
(a) Adsorption mechanism, (b) Nanocellulose surface chemistry.

The mechanisms that make adsorptive desulphurization viable at ambient conditions with low energy consumption and no loss of octane value include π-complexation, reactive adsorption, van der Waals, and electrostatic interactions [40,45,120]. Also, the functional groups (surface chemistry) that have contributed to the application of nanocellulose for selective desulphurization of gasoline are the carboxyl group (-COOH), a hydroxyl group (-OH), carbonyl group (-CO), and aldehyde group (-CHO) [121]. The adsorption mechanism and surface chemistry (functional groups) are shown in Fig. 7(a) and (b), respectively.

Moreover, selective adsorption occurs during the acid-base reaction of sulphur and metal, leading to *S*-adsorbent (*S*-M) interaction [122]. The adsorptive mechanism of π-complexation occurs when cations form σ-bonds with s-orbitals of sulphur while back-donating their d-orbitals improves the electron density to the anti-bonding π-orbitals of the sulphur rings [45]. The metal's vacant s-orbitals and the availability of the high-electron density d-orbitals needed for back-bonding created a strong π-complexation bonding [40]. Although biomass-based adsorbents have unique properties for desulphurization, to achieve the sustainability of adsorptive desulphurization of petroleum distillates using green adsorbents, there is a need to overcome the challenges associated with the utilization of nanocellulose crystal extracted from waste biomass through green isolation techniques for adsorptive desulphurization.

## 8 Challenges and outlook of green isolation techniques for nanocellulose crystal as adsorbent for desulphurization of liquid fuels

The main challenge with the nanocellulose crystals isolation from lignocellulosic waste biomass is the need to carry out a pretreatment process on the waste before the isolation of the process to solubilize the hemicellulose and lignin components of the lignocellulosic waste. The pretreatment process is critical in valorizing lignocellulosic waste to improve its digestibility for efficiently isolating nanocellulose crystals. However, the conventional pretreatment process involving chemicals is unsustainable due to the generation of harmful by-products, environmental pollution due to solvent waste, toxicity, elevated energy demand, and safety concerns [123]. Thus, the green isolation techniques that will effectively substitute the conventional pretreatment of lignocellulosic waste are required to resolve the associated challenges with the traditional approach.

Different challenges still affect the green isolation techniques for nanocellulose crystal, notably when used on an industrial scale. These challenges include technical concerns for designing specific and efficient green isolation techniques for nanocellulose crystal extraction from lignocellulosic biomass waste and surface modification of nanocellulose crystal [124]. Another issue is high running costs, especially for the principal green isolation approaches that use aqueous ionic liquids and energy-intensive liquid hot water treatments. A significant amount of water and energy are needed for the liquid hot water pretreatment procedure. Also, the ionic liquids pretreatment technique involves many expensive ionic liquids, and the solution becomes viscous with hemicellulose and lignin, making the solution challenging for further isolation [109]. Therefore, the green isolation techniques require more studies to improve the methods, focus on cheaper synthesis approaches for better industrial applications, upscale the process, and optimize the process parameters. Moreover, the green isolation techniques for nanocellulose crystal might be regarded as the outlook for sustainable treatment of lignocellulosic waste as they do not produce hazardous by-products or inhibitor compounds, and the nanocellulose crystal yield is relatively high.

The nanocellulose crystal as an adsorbent for effectively removing sulphur compounds from fuels was developed as a substitute for the expensive technology and energy-intensive activated carbon-based adsorbents. However, the nanocellulose crystal has been proposed as an excellent adsorbent due to its large surface area, crystallinity property, adsorption capacity, and affinity towards organic contaminants [125]. Nevertheless, it must be specially designed to improve interactions for the desired result and selectively adsorb organic sulphur compounds from petroleum distillates. Furthermore, the adsorptive desulphurization process and the isolation of nanocellulose crystals from lignocellulosic waste have been mainly investigated on a laboratory scale. Therefore, the challenge with the application of nanocellulose crystal for the adsorptive removal of organic sulphur compounds from liquid fuels includes the industrial up-scaling of the process, selectivity, and affinity for aromatic sulphur organic compounds, cost implication associated with the isolation techniques of nanocellulose crystal and environmental impact of modified nanocellulose crystal. This review discovered that technical concerns and operating costs are the main challenges affecting the green isolation technique for extracting nanocellulose crystals. However, the challenges can be overcome with rigorous and pilot-scale investigations to industrialize the designs and construct economically and environmentally feasible green isolation of nanocellulose crystal process and desulphurization of petroleum distillates. Moreover, artificial intelligence technologies will enhance the prediction and optimization of nanocellulose crystals as an adsorbent for efficient desulphurization of refinery products.

## 9 Conclusions

The incorporation of 4IR technologies for effective adsorptive desulphurization can assist in achieving the globally acceptable sulphur level in refinery products and enhance sustainable development goals. Applying artificial intelligence or machine learning tools to predict and optimize desulphurization operating conditions would reduce process time, save operation costs, and minimize energy consumption. The initial sections of this review broadly discussed the synergistic relationship between nanotechnology and artificial intelligence in adsorptive desulphurization compared to other desulphurization techniques. The study realized that technical concerns and operating costs, among others, are the main challenges affecting the optimization of the desulphurization process using machine learning tools. The middle sections focused on applying artificial intelligence tools in waste valorization to promote environmental sustainability and optimize the green isolation operating conditions. Likewise, the adsorption capacity of nanocellulose crystal isolated from waste biomass can be increased by optimizing the process parameters and enhancing the adsorbent suitability to improve the desulphurization efficiency. Also, the last sections highlighted the utilization of waste biomass-derived adsorbents as suitable alternatives to non-green adsorbents in adsorptive desulphurization. In addition, this review identified that the biomass-based nanocellulose crystal through the green isolation technique had not been widely applied as an adsorbent for the adsorptive desulphurization of refinery products. In future, the 4IR technologies for adsorptive desulphurization would combine nanotechnology and artificial intelligence to boost the production of cleaner fuels, have substantial socio-economic benefits, and promote environmental sustainability. Another prospect is the recommended application of two or more artificial intelligence tools in conjunction with each other for predicting and optimizing desulphurization operating conditions.

## Funding

This work is based on the research supported by the 10.13039/501100001321SASOL–National Research Foundation (NRF) of South Africa (Grant Number 138620) and 10.13039/501100001321National Research Foundation (NRF) of South Africa Scholarship (Grant Number: PMDS22062024929) awarded to Oluwagbenga A. Olawuni for his PhD degree programme.

## CRediT authorship contribution statement

**Oluwagbenga A. Olawuni:** Methodology, Writing - original draft, Investigation, Formal analysis. **Olawumi O. Sadare:** Writing - review & editing, Supervision, Conceptualization. **Kapil Moothi:** Writing - review & editing, Supervision, Project administration, Funding acquisition.

## Declaration of competing interest

The authors declare that they have no known competing financial interests or personal relationships that could have appeared to influence the work reported in this paper.
